# Room Temperature Electrically Detected Nuclear Spin Coherence of NV Centres in Diamond

**DOI:** 10.1038/s41598-020-57569-8

**Published:** 2020-01-21

**Authors:** H. Morishita, S. Kobayashi, M. Fujiwara, H. Kato, T. Makino, S. Yamasaki, N. Mizuochi

**Affiliations:** 1 Institute for Chemical Research, Kyoto University, Gokasho, Uji, Kyoto 611-0011 Japan; 2 CREST, Japan Science and Technology Agency, Kawaguchi, Saitama, 332-0012 Japan; 3 Energy Technology Research Institute, National Institute of Advanced Industrial Science and Technology (AIST), Tsukuba, Ibaraki, 305-8568 Japan

**Keywords:** Electronic and spintronic devices, Electronic devices, Sensors, Quantum information

## Abstract

We demonstrate electrical detection of the ^14^N nuclear spin coherence of NV centres at room temperature. Nuclear spins are candidates for quantum memories in quantum-information devices and quantum sensors, and hence the electrical detection of nuclear spin coherence is essential to develop and integrate such quantum devices. In the present study, we used a pulsed electrically detected electron-nuclear double resonance technique to measure the Rabi oscillations and coherence time (*T*_2_) of ^14^N nuclear spins in NV centres at room temperature. We observed *T*_2_ ≈ 0.9 ms at room temperature, however, this result should be taken as a lower limit due to limitations in the longitudinal relaxation time of the NV electron spins. Our results will pave the way for the development of novel electron- and nuclear-spin-based diamond quantum devices.

## Introduction

Nuclear spins in a semiconductor have a long coherence time (*T*_2_) due to the good isolation from environmental noise^1–7^. Therefore, they are candidates for quantum memories in quantum-information devices and quantum sensors. Using nuclear spins (e.g., nitrogen and carbon) in diamond for quantum memories, highly sensitive magnetic sensors^8–10^, quantum repeaters^11^, quantum registers^12,13^, etc., have been demonstrated at room temperature. In these demonstrations, the detection of nuclear spin coherence is essential. Nuclear spin coherence can be detected via the electron spins of nitrogen-vacancy (NV) centres, which also have a long *T*_2_ at room temperature^14–16^. NV electron spins can be detected by optical techniques^17^ and electrical techniques^18–20^. The electrical technique is an important technology for developing and integrating quantum devices. Furthermore, a theoretical model predicts that its detection sensitivity is approximately three times higher than that of the optical technique^19^. While the photoelectrical detection of the electron spin coherence of an ensemble of NV centres^19,20^ and photoelectrical coherent spin-state readout of single NV centres^21^ at room temperature has been demonstrated, the direct electrical detection of nuclear spin coherence remains challenging. This is because nuclear spins are well isolated from the environment and the interactions between nuclear spins and current are very weak. Thus, we focus on an electrically detected electron-nuclear double resonance (EDENDOR) technique to demonstrate room-temperature electrical detection of nuclear spin coherence.

The first EDENDOR measurement was demonstrated by Stich and collaborators for phosphorus (P) donors in silicon at 4.2 K^22^. After this demonstration, two other groups independently demonstrated pulse EDENDOR measurements of P-donors in silicon^23–26^. They measured Rabi oscillations and *T*_2_ of P-donor nuclear spins from 3.5 to 5 K. Furthermore, pulsed EDENDOR measurements of proton nuclear spins in organic semiconductors have been demonstrated^27^. Proton nuclear spin resonances have been measured at room temperature, but Rabi and *T*_2_ measurements of proton nuclear spins have not been reported yet. To the best of our knowledge, there have been no demonstrations of room-temperature electrical detection of nuclear spin coherence in diamond or any other materials.

**Figure 1:**
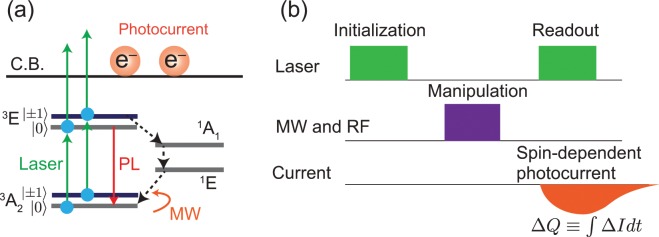
(**a**) Schematic of the EDENDOR measurements of NV centres in diamond. (**b**) Process for the EDENDOR measurements.

The EDENDOR signals of ^14^N nuclear-spin coherence are observed by measuring the change in the electrically detected electron-spin echo (ESE) intensity of the NV centres. Here, the technique of electrical detection of magnetic resonance signals is called electrically detected magnetic resonance (EDMR). The EDMR of the NV centres measures the photocurrent change due to electron-spin resonances of the NV centres^18–21^. The photocurrent can be generated under illumination by a 532-nm laser via a two-photon ionisation process, depicted in Fig. 1(a). The figure shows that the |±1〉 NV electron spin at the ^3^*E* state has a transition probability to the long-lived (~220 ns) metastable ^1^*E* states, where |*m*_*s*_〉 describes an NV electron spin. This causes the photocurrent to decrease due to a magnetic resonance transition from |0〉 to |±1〉 after the optical initialisation to |0〉^18^. Based on this mechanism, this study measures the electron and nuclear spin coherences with the pulse sequence depicted in Fig. 1(b). The details of the pulse sequence are as follows. (1) A 532-nm laser is used to initialise the NV electron spins to |0〉. (2) The electron and/or nuclear spins are manipulated by microwave (MW) and/or radiofrequency (RF), respectively. (3) The final laser is used to generate the photocurrent via two-photon ionisation, which contains the EDMR and EDENDOR signals. They are observed as a transient response, which is depicted in Fig. 1(b). Hence, this study defines the EDMR and EDENDOR intensities (∆*Q*) as the change in the photocurrent (∆*I*) integrated by the time during which the photocurrent is changing, which is described as ∆*Q* ≡ ∫∆*Idt*^27,28^. Thus, the unit of the intensities of EDMR and EDENDOR can be described by coulomb (C)^27,28^.

## Result

### Pulsed EDMR spectrum of ensemble of NV centre

**Figure 2:**
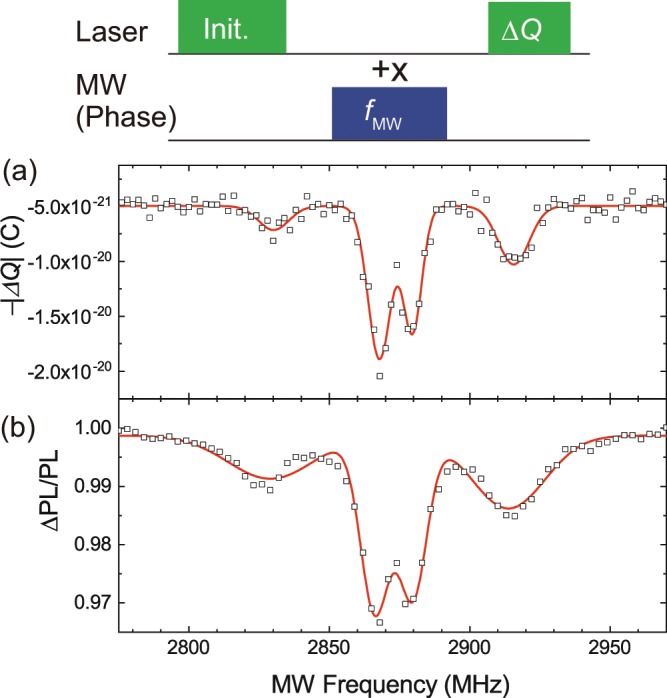
(Top) Pulse sequence for pEDMR and pODMR measurements. +*x* indicates as the phase of MW pulse. (**a**) pEDMR spectrum. (**b**) pODMR spectrum.

Initially, we performed a pulsed EDMR (pEDMR) measurement with the pulse sequence depicted in the top of Fig. 2. We used 30 mW of laser power and an input MW power of 10 mW at a static magnetic field of ~10 G approximately along the [111] direction of the diamond crystal. Figure 2(a) shows the EDMR intensity (∆*Q*) as a function of MW frequency, showing that four signals appeared. To analyse the observed spectrum, we measured the pulsed ODMR (pODMR) spectrum shown in Fig. 2(b) with the same conditions as the pEDMR measurements. The pEDMR spectrum has different linewidths than the pODMR spectrum. This may be due to the input laser power. While the pEDMR measurements require the illumination of the 30-mW laser to generate the photocurrent, the illumination of the 30-mW laser is strong enough to make broad linewidths in the pODMR signals. However, both spectra have the same resonant frequencies, and hence the NV electron spin resonance signals are observed via the electrical technique.

### Rabi oscillations of NV electron spins

**Figure 3:**
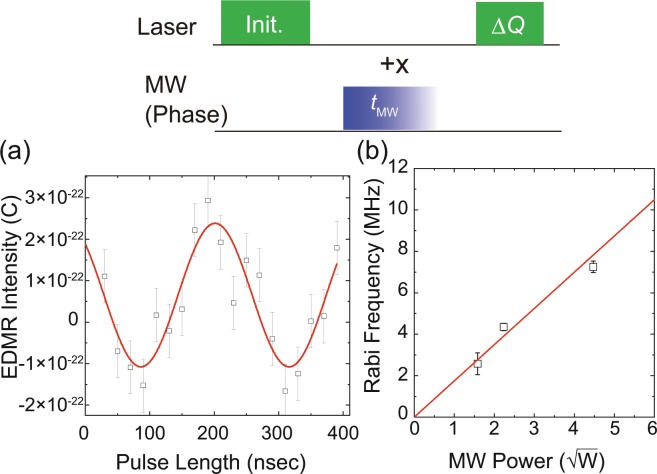
(Top) Pulse sequence of electron-spin Rabi oscillation measurements. +*x* indicates the phase of MW pulse. (**a**) Electrically detected electron-spin Rabi oscillation. (**b**) Rabi frequencies as a function of the square root of MW power.

The top of Fig. 3 shows the pulse sequence for the electron-spin Rabi oscillations. After the initialisation of the NV electron spins by the first laser pulse, we measured ∆*Q* under the application of the last laser as a function of the length of the resonant MW pulse (*t*_MW_). Here, we set the MW frequency to 2916 MHz, corresponding to the transition between |0〉 and |+1〉, and its input power to 5 W. The result is shown at the bottom of Fig. 3(a). The solid line in Fig. 3(a) shows the curve fitting result with a sinusoidal curve. We observed an oscillation frequency of ~4.4 MHz. Next, we measured the oscillation frequencies with three different input MW powers, as depicted in Fig. 3(b). The figure plots the oscillation frequencies as a function of the square root of the input MW power. The observed data can be fitted by a linear function fixing the intercept at zero, and hence we observed Rabi oscillations of NV electron spins and the Rabi frequencies could be controlled by changing the input MW power.

### EDENDOR spectrum of ^14^N nuclear spins

**Figure 4:**
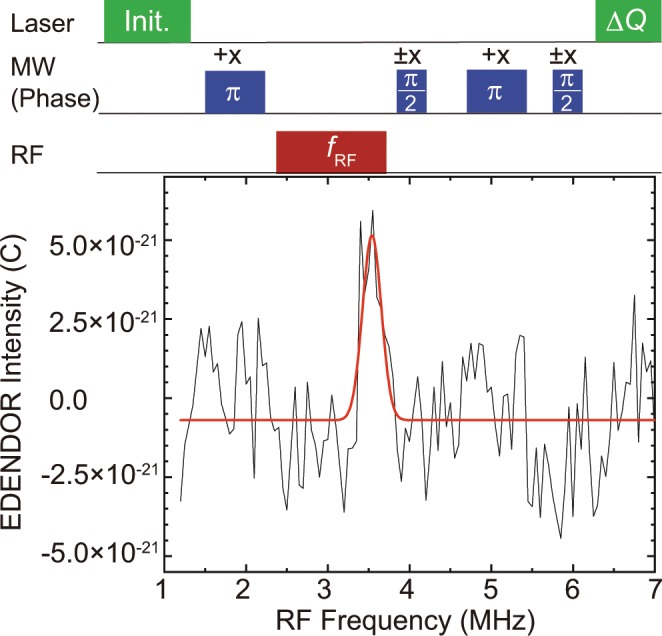
Pulse sequence (top) and the result (bottom) of an electrically detected ENDOR spectrum. ±*x* indicate the phase of MW pulse.

To measure the nuclear magnetic resonance of ^14^N nuclear spins, we used the ENDOR technique (The detail of the ENDOR is explained in Supplementary Information). This enables the detection of a nuclear magnetic resonance (NMR) signal via a change in the ESE intensity based on the polarisation transfer between the electron and nuclear transitions using the pulse sequence depicted in the top of Fig. 4^24^. First, an MW *π*-pulse is applied to the transition between |0〉 to |+1〉 after illumination by a pulsed laser. This *π*-pulse can generate hyperfine coupling between NV electron spins and ^14^N nuclear spins and the polarisation between |+1, 0〉 and |+1, +1〉, where |*m*_*s*_*,m*_*I*_〉 are electron and nuclear spins, respectively^29–31^. Then, the RF pulse is applied. Finally, ∆*Q* was measured by applying a Hahn echo sequence and the following laser pulse. We observed EDENDOR spectra by measuring ∆*Q* as a function of the irradiated RF frequency.

Setting the MW frequency to 2916 MHz, the input MW power to 5 W, and the input RF power to 5 W, we observed the spectrum shown in Fig. 4. The observed data can be fitted with the Gaussian function shown by the solid red line in Fig. 4. The curve fitting result shows that we observed a resonance frequency of 3.5 MHz. Using equation (S1) in the Supplementary Information, we can estimate that the observed resonance frequency corresponds to the transition between |+1, 0〉 and |+1, +1〉. Thus, we focus on the transition with the resonance frequency of 3.5 MHz between |+1, 0〉 and |+1, +1〉 in the following measurements of the Rabi oscillations and the coherence time of the ^14^N nuclear spins in this study.

### Rabi oscillations of ^14^N nuclear spins

**Figure 5:**
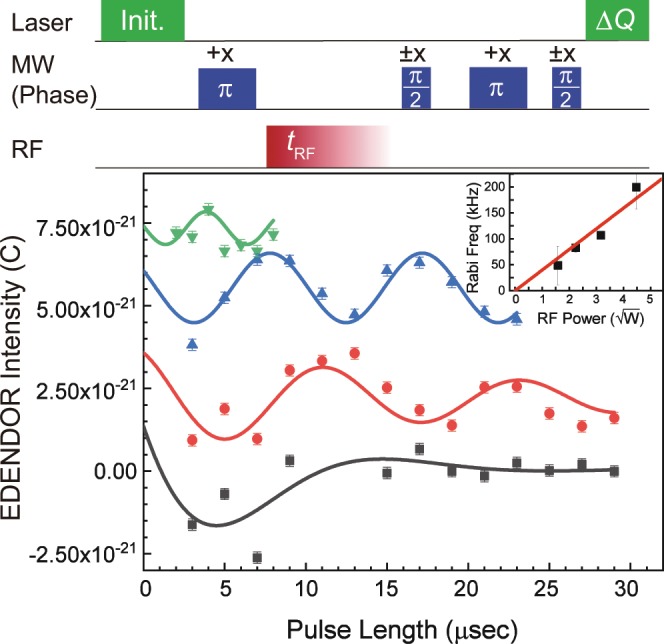
Pulse sequence (top) and the result (bottom) of an electrically detected nuclear Rabi oscillation. ±*x* indicate the phase of MW pulse. (Inset) Rabi frequencies as a function of the square root of RF power.

The top of Fig. 5 shows the pulse sequence for the measurements of the ^14^N nuclear-spin Rabi oscillations. The sequence shows that we measured ∆*Q* as a function of the length of the RF pulse. Here, we set the MW frequency to 2916 MHz, the input MW power to 5 W, and the RF frequency to 3.5 MHz. The results of the electrically detected nuclear-spin Rabi measurements with four different input RF powers are shown in the bottom of Fig. 5. The red, black, blue, and green points correspond to the results with input RF powers of ~2.5, 5, 10, and 20 W, respectively. The observed oscillations are fitted by sinusoidal curves, which are shown as solid lines in the bottom of Fig. 5. The results of the curve fittings showed that all Rabi oscillations have the same phase offset which depends on the polarisation of ^14^N nuclear spin after applying the MW *π* pulse (The detail of the pulse sequence of ENDOR is explained in Supplementary Information). Furthermore, the oscillation frequencies observed by the curve fittings are plotted as a function of the square root of the input RF power in the inset of Fig. 5. The plots are fitted well by a linear function with the intercept at zero, as shown by the solid line, which certifies that the observed oscillations correspond to Rabi oscillations between |+1, 0〉 and |+1, +1〉. Hence, the Rabi oscillations of the ^14^N nuclear spins can be observed with EDENDOR at room temperature.

### Echo decay of ^14^N nuclear spins

**Figure 6:**
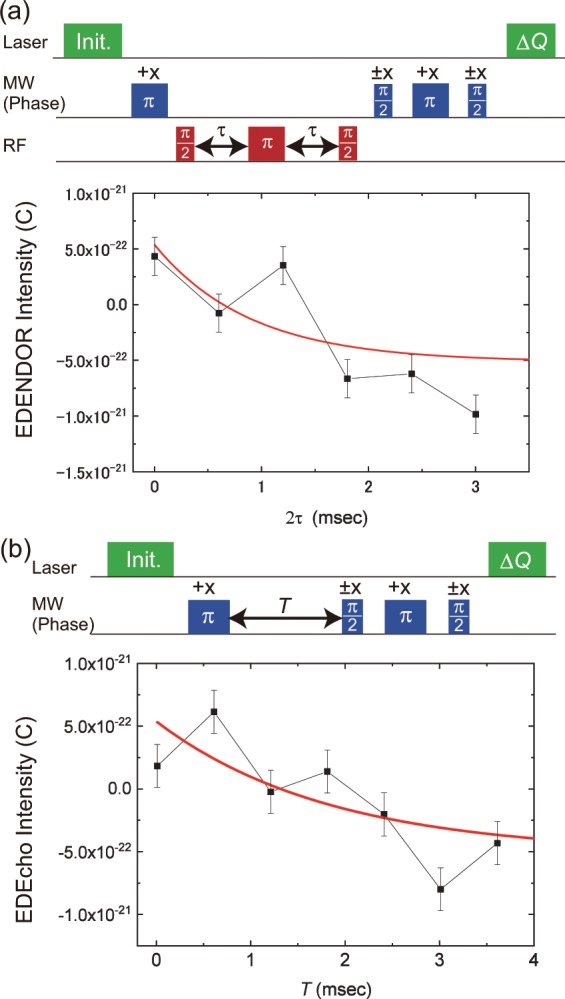
(**a**) Pulse sequence (top) and the result (bottom) of an electrically detected ${T}_{2}^{(n)}$ measurement of the ^14^N nuclear spins in NV centres. ±*x* indicate the phase of MW pulse. (**b**) Pulse sequence (top) and result and electrically detected ${T}_{1}^{(e)}$ measurement of the NV electron spins (bottom). ±*x* indicate the phase of MW pulse.

The top of Fig. 6(a) shows the pulse sequence for a ^14^N nuclear spin (${T}_{2}^{(n)}$) measurement. It can also be modified based on the EDENDOR sequence to measure ${T}_{2}^{(n)}\,$^25,26,32^. A nuclear-spin Hahn echo sequence was added between the first MW *π*- and second MW *π*/2-pulse, allowing the nuclear spin echo intensity to be measured via the change in the ESE intensity. We set the input RF power to 10 W and the other experimental conditions were the same as those for electrically detected nuclear spin Rabi oscillations. Then, we measured ∆*Q* as a change of the ESE intensity as a function of the freely evolving time of 2*τ*. The bottom of Fig. 6(a) shows the result of the ${T}_{2}^{(n)}$ measurement. The experimentally observed plots are fitted with an exponential curve described by the solid line in Fig. 6(a) fixing the echo amplitude at half the Rabi amplitude. Consequently, ${T}_{2}^{(n)}$ ≈ 0.9 (5) ms. Hence, we successfully observed ${T}_{2}^{(n)}$ with the EDENDOR technique at room temperature.

## Discussion

We here discuss the results of the ${T}_{2}^{({\rm{n}})}$ measurement with the modified EDENDOR sequence. After the application of the first MW *π*-pulse in the sequence of the ${T}_{2}^{({\rm{n}})}$ measurement depicted in Fig. 6(a), the NV electron spins relax back to their thermal equilibrium with a time constant defined as the longitudinal relaxation time ${T}_{1}^{({\rm{e}})}$. Thus, the observed ${T}_{2}^{(n)}$ may be limited by ${T}_{1}^{({\rm{e}})}$ in our experiments. We measured ${T}_{1}^{({\rm{e}})}$ with the EDMR technique. The pulse sequence for a ${T}_{1}^{({\rm{e}})}$ measurement is shown in the top of Fig. 6(b). It is the same as the sequence for ${T}_{2}^{(n)}$ measurements, without the train of RF pulses. Using the same experimental conditions as those for the ${T}_{2}^{(n)}$ measurement, except for the irradiation by the RF pulses, we measured ∆*Q* as the change in the ESE intensity as a function of *T*, which is the interval between the first MW *π*- and second MW *π*/2-pulses. In the bottom of Fig. 6(b), the experimentally observed data points were fitted with the exponential curve described by the solid line. This gave ${T}_{1}^{(e)}\approx$ 1.8(6) ms, which is slightly shorter than the reported ${T}_{1}^{({\rm{e}})}$^12^, which may be due to the additional decay rate into |0〉 due to laser leakage through an acousto-optic modulator (AOM) and/or noises in the magnetic and electric fields from P-donors^33,34^ and/or from a dark electrical current in the absence of the laser illumination. Myers *et al*.^33^ showed that the electrical field from the diamond surface noise influences the ${T}_{1}^{({\rm{e}})}$ of the NV electron spins. Since the electron carriers released from the P-donors and the positive charge of the P-donors after the release generates the electric field, the ${T}_{1}^{({\rm{e}})}$ of the NV electron spins may become short in the highly P-doped diamond layer. Moreover, the ${T}_{1}^{({\rm{e}})}$ measurement was performed in the presence of a dark current (The details of a dark current are explained in Supplementary Information). The dark current produces magnetic fluctuations^34^, which may shorten ${T}_{1}^{({\rm{e}})}$.

Finally, we compare the observed ${T}_{1}^{({\rm{e}})}$ with ${T}_{2}^{(n)}$. When the ${T}_{2}^{(n)}$ decay is affected only by ${T}_{1}^{({\rm{e}})}$, the theory expects ${T}_{2}^{(n)}=\frac{3}{2}{T}_{1}^{(e)}$^10^. However, the observed ${T}_{2}^{(n)}$ is slightly shorter than ${T}_{1}^{({\rm{e}})}$. When we consider the influence of laser leakage through the AOM accompanied by additional decay rate into $|0\rangle ,{T}_{2}^{(n)}$ might become shorter than $\frac{3}{2}{T}_{1}^{(e)}$, as discussed in ref. ^10^. Thus, precise control of the concentration of P-donors and the upgrade of the EDENDOR spectrometer are important in order to observe long ${T}_{1}^{({\rm{e}})}$ and ${T}_{2}^{(n)}$ by the EDENDOR technique.

In summary, we have demonstrated electrical detection of the Rabi oscillations and ${T}_{2}^{(n)}\,$of the ^14^N nuclear spin coherence of an ensemble of NV centres in diamond at room temperature. Using a self-built EDENDOR spectrometer, we observed a signal at ~3.5 MHz, which is the ENDOR frequency of the ^14^N nuclear spins. This frequency was used to demonstrate electrically detected nuclear-spin Rabi oscillations and ${T}_{2}^{(n)}$ measurements of the ^14^N nuclear spins at room temperature. We observed ${T}_{2}^{(n)}\approx$ 0.9 ms. This study should contribute to the development of future electron- and nuclear-spin based diamond quantum devices.

## Method

### Sample preparation

In this study, we used an ensemble of NV centres in a P-doped *n*-type diamond layer. This sample has two characteristics: (1) a negative charge state of the NV centre (NV^−^) in the P-doped *n*-type diamond that is stable even under 532-nm laser illumination^35^ and (2) the highly P-doped *n*-type diamond has high electrical conductivity^36^. The ensemble of NV centres in the *n*-type diamond was prepared via the following processes. A P-doped *n-*type diamond layer (10-*µ*m thick) was synthesised on a type IIa (001) diamond substrate by chemical vapour deposition (CVD)^36^. The *n*-type diamond layer has a P-donor concentration of ~10^18^ cm^−3^. The ensemble of NV centres was made by ^14^N^+^-ion implantation (dose: 1 × 10^15^ cm^−2^) with a kinetic energy of 350 keV, followed by annealing at 1000 °C for 1 hour under vacuum. We estimated the concentration of NV centres in the detection volume of the self-built EDENDOR spectrometer. The detection volume of the confocal microscope used as a laser illumination unit in the EDENDOR spectrometer can be estimated as 2 × 10^−12^ cm^3^ (Details are explained in Supplementary Information). When the fluorescence from the NV centre is proportional to the concentration of NV centres, we can estimate the concentration of NV centres of 1 × 10^15^ cm^−3^ (2 × 10^3^ NV centres in the spot size of the focused laser beam). After generating the ensemble of NV centres, interdigital contacts with ~2-*µ*m gaps were fabricated on the *n*-type diamond layer to detect photocurrent changes, by the following three steps: (1) electron-beam lithography, (2) deposition of Ti(30 nm)/Pt(30 nm)/Au(100 nm) multilayers, and (3) annealing at 420 ^◦^C for 30 min under an argon atmosphere. Details of the electrical characteristics of the electrical contacts are given in Supplementary Information.

### Self-built EDENDOR spectrometer

**Figure 7:**
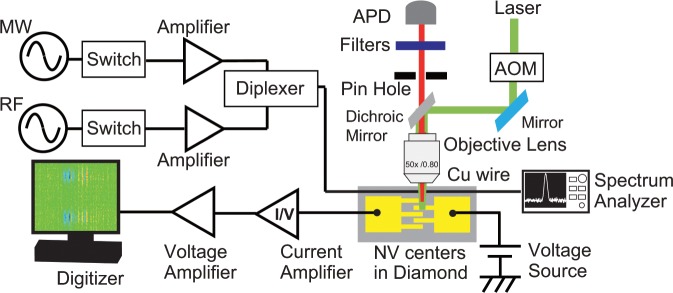
Schematic of the self-built EDENDOR spectrometer.

Figure 7 shows a self-built EDENDOR spectrometer (Details are explained in Supplementary Information). This spectrometer consists of (1) a laser illumination unit of a confocal microscope with a 532-nm laser to generate a photocurrent from an ensemble of NV centres, (2) a microwave (MW) and radio-frequency (RF) irradiation unit to manipulate the NV electron and ^14^N nuclear spins, and (3) a photocurrent detection unit. Using the laser illumination unit, the laser illuminates the ensemble of NV centres, focused by an objective lens with an NA of 0.8. Then, a photocurrent is generated from the NV centres. The NV centres also emit photons under the laser illumination, which are detected by an avalanche photodiode (APD) after passing through a pinhole and a filter (633-nm long-pass filter). In this study, the APD is used to fix the position of the illumination spot in a place between the electrical contacts by monitoring the photons from the NV centres. MW and RF are combined with a frequency diplexer and irradiated to the NV centres with a ~50-*µ*m copper wire. The irradiated MW and RF frequencies and powers are measured by a spectrum analyser during the EDMR and EDENDOR measurements. The change of photocurrent is measured under the application of a constant voltage of 8 V. The change of photocurrent is converted into a change of voltage by a current amplifier. Then, it can be measured by a digitiser on a personal computer after amplification by a voltage amplifier. In this study, we measured ~2 × 10^3^ NV centres in the EDMR and EDENDOR measurements and observed their signals from 10^7^ or more repetitions. A phase cycling technique is applied to subtract the artefact noises due to on- and off-resonant MW and RF contributions and laser-power fluctuations from the EDMR and EDENDOR signals^27,32,37^ (The details of the phase cycling are explained in Supplementary Information). Note that the phases of the MW pulses are indicated by ±*x* on the MW pulses shown in Figs. 2–6.

## Supplementary information

Supplementary Information.

## Data Availability

The data that support the findings of this study are available from the corresponding author upon reasonable request.
