# Supplementary material for: Room Temperature Electrically Detected Nuclear Spin Coherence of NV Centres in Diamond
Source: Sci Rep. 2020 Jan 21;10:792. doi: 10.1038/s41598-020-57569-8 (PMC6972904; doi:10.1038/s41598-020-57569-8)
Supplement: Supplementary file 1 — Supplementary Information. [file 41598_2020_57569_MOESM1_ESM.pdf]

Supplementary information

Room Temperature Electrically Detected Nuclear Spin Coherence of NV Centre in Diamond

H. Morishita,[1,2,*] S. Kobayashi,[1,2] M. Fujiwara,[1,2] H. Kato,[2,3] T. Makino,[2,3] S. Yamasaki,[2,3] and N. Mizuochi[1,2,†]

[1]*Institute for Chemical Research, Kyoto University, Gokasho, Uji, Kyoto 611-0011, Japan*

[2]*CREST, Japan Science and Technology Agency, Kawaguchi, Saitama 332-0012, Japan*

[3]*Energy Technology Research Institute, National Institute of Advanced Industrial Science and Technology (AIST), Tsukuba, Ibaraki 305-8568, Japan*

NV Centre Coupled to a ^{14}N Nuclear Spin

The Hamiltonian for an NV electron spin coupled with a ^{14}N nuclear spin under a static magnetic field (B_0)[s1] is:

$$\mathcal{H} = D_{\text{gs}}\left[S_z^2 - \frac{1}{3}S(S+1)\right] + g_e\mu_B B_0 S_z$$

$$+g_n\mu_n B_0 I_z + A_{\parallel}S_z I_z + A_{\perp}(S_x I_x + S_y I_y) + P\left[I_z^2 - \frac{1}{3}I(I+1)\right] \quad \text{(S1)}$$

The first and second terms are described as zero-field splitting and Zeeman interaction of NV electron spins, where $D_{\text{gs}} \sim$ 2.87 GHz, S_z, g_e ~ 2.003, and μ_B are a zero-field splitting parameter, the z component of the NV electron spin (S), the g-factor of the NV electron spin, and the Bohr magnetron, respectively. The third term is described as Zeeman interaction of ^{14}N nuclear spin, where $g_n \sim$ 0.404 and μ_n are the g-factor of the ^{14}N nuclear spin and nuclear magneton, respectively[s2]. The fourth and fifth terms are described as axial ($A_{\parallel} \sim$ −2.1 MHz) and non-axial hyperfine interaction ($A_{\perp} \sim$ −2.7 MHz) with the ^{14}N nuclear spin, respectively[s3]. The sixth term is described as the quadrupole interaction of the ^{14}N nuclear spin ($P \sim$ −5.0 MHz)[s3]. I_x, I_y, and I_z are x, y, and z components of the ^{14}N nuclear spin (I), respectively.

Self-built EDENDOR Spectrometer

A self-built EDENDOR spectrometer consists of the following three units; 1) laser illumination unit, 2) microwave (MW)- and radiofrequency (RF)-irradiation unit, and 3) photocurrent detection unit depicted in Fig. 7 of the main text. The confocal laser microscope with a 532-nm laser works as the laser illumination unit. The 532nm laser was pulsed by an acousto-optic modulator (AOM). After the pulsed laser is reflected by a dichroic mirror, it illuminates the ensemble of the NV centre focused by an objective lens with NA of 0.8. When the objective lens has NA of 0.8 and laser has a wavelength of 532 nm, we can estimate the detection volume of our confocal laser microscope of 2×10^{-12} cm^3 [s4]. Using the laser illumination unit, the photocurrent from the NV centres is generated under the laser illumination. Moreover, photons emitted from the NV centres were detected by an avalanche photodiode (APD) after passing a pinhole with a diameter of ~ 30 μm and two filters (an 835-nm short-pass filter and a 633-nm long-pass filter). In this study, the APD was used to fix the position of the illumination spot in a place depicted in the white circle in Fig. S1(b). In the MW- and RF-irradiation unit, MW and RF generated by two high-frequency oscillators are pulsed by frequency switches. After the amplification of pulsed MW and RF with MW and RF amplifiers, they are combined with a frequency diplexer. Then, they are irradiated to the NV centres by a copper wire with a diameter of ~50 μm. Here we used a spectrum analyser to measure the irradiated MW and RF frequencies and powers during EDMR and EDENDOR measurements. We measure a change of photocurrent under the application of a constant voltage (SRS SIM928) with the photocurrent detection unit. In the photocurrent detection unit, the change of photocurrents is converted a change of voltage by a current amplifier (FEMTO DHPCA-100). Then, the change of voltage can be measured by a digitiser (Gage Razor CSE1621) on a personal computer after the amplification with a voltage amplifier (FEMTO DHPVA-201).

Evaluation of Electrical Contacts

Figure S1(a) and S1(b) show the photograph and PL later-scan image with laser power of 15 μW of electrical contacts for the EDMR and EDENDOR measurements. When we set the laser power to 30 mW and the laser spot fixed to the position depicted in the white circle in Fig. S1(b), we measured current-voltage characteristics of the electrical contacts with and without laser illumination. The blank and filled points in Fig. S1(c) show the current-voltage characteristics with and without laser illumination, respectively. It shows that dark current which is current in the absence of the laser illumination flows in the diamond and photocurrent generates under the laser illumination.

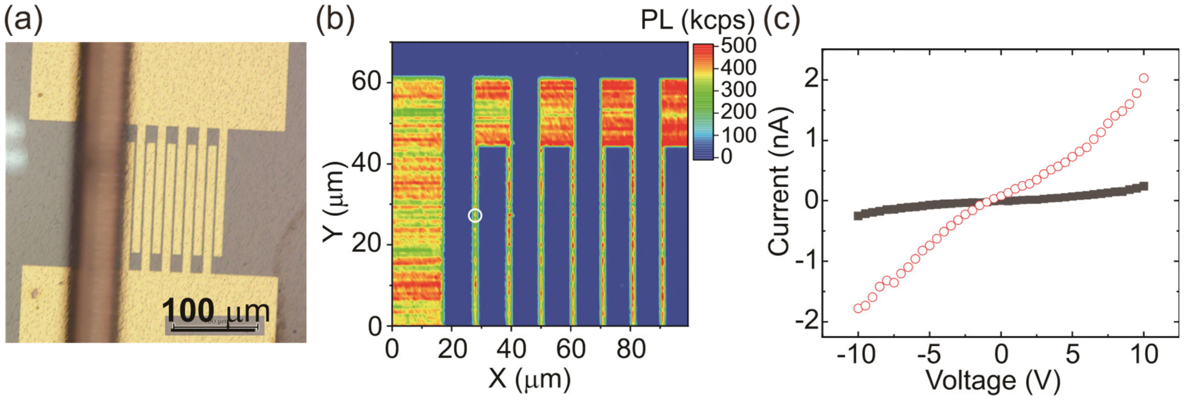

FIG. S1. (a) Photograph of electrical contacts with MW antenna. (b) PL laser-scan image of electrical contacts. (c) IV characteristics of electrical contacts. The hollow and filled points show the IV characteristics with and without laser illumination, respectively.

Phase Cycling

A phase cycling technique is used to subtract on- and off-resonant MW and RF contributions to and fluctuation of laser power to magnetic resonance signals. The phases of MW pulses are indicated by the ±*x* on the MW pulses in pulse sequences depicted in Figs. 4, 5, and 6 of the main text. In order to explain such a phase cycling technique, we discuss the polarities of Hahn-echo signals with four different phase cycling configurations depicted in Fig. S2. We observe positive echo signals in the cases of (i) and (iii) of Fig. S2 and negative echo signals in the case of (ii) and (iv) of Fig. S2[s5–s7]. On the other hand, the polarities of MW and RF currents due to the irradiation of on- and off-resonant MW and RF fields and photocurrent noise induced by the laser power fluctuation do not change with the above phase cycling configurations. Thus, we observe just magnetic resonance signals to add the echo signals with the sequences of (i) and (iii) to the sum of the magnetic resonance signals and to subtract the echo signals with the sequences of (ii) and (iv) from one [s5–s7].

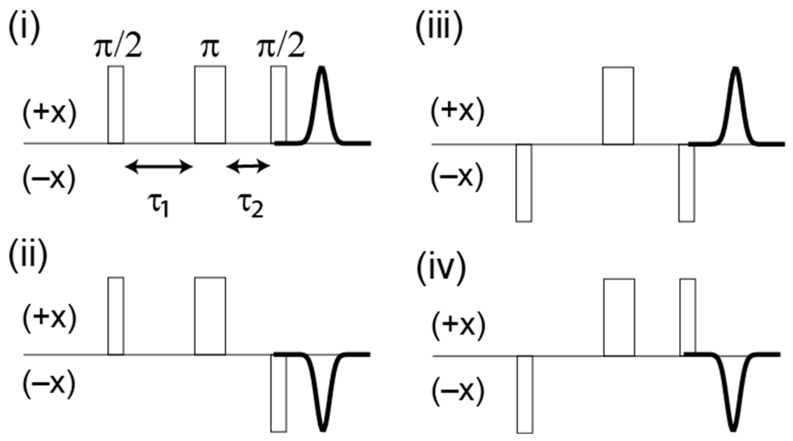

FIG. S2. Electron-spin Hahn echo measurements with four different phase cycling configurations.

ENDOR

We consider the quantum system of an electron spin (S = 1/2) coupled with a nuclear spin (I = 1/2). The top of Fig. S3 shows the pulse sequence of the ENDOR[s5], and the bottom of Fig. S3 shows energy levels of the system, where $|\pm,\pm\rangle$ are defined as the electron and nuclear spins, respectively. After the system is initialised to $|--\rangle$ which is depicted filled rectangular, the MW π pulse inverts the polarisation of the transition between $|--\rangle$ and $|+-\rangle$ (bottom left of Fig. S3). Next, the resonant RF π pulse is applied to the transition between $|+-\rangle$ and $|++\rangle$. Then, its polarisation is inverted (bottom centre of Fig. S3). Finally, we measured the echo of the transition between $|--\rangle$ to $|+-\rangle$ (bottom right of Fig. S3). In this situation, we do not observe any echo signals. Such a change of the echo signal means a nuclear-magnetic-resonance transition between $|+-\rangle$ and $|++\rangle$ occurs. Therefore, we can observe the nuclear magnetic resonance signals with the ENDOR sequence.

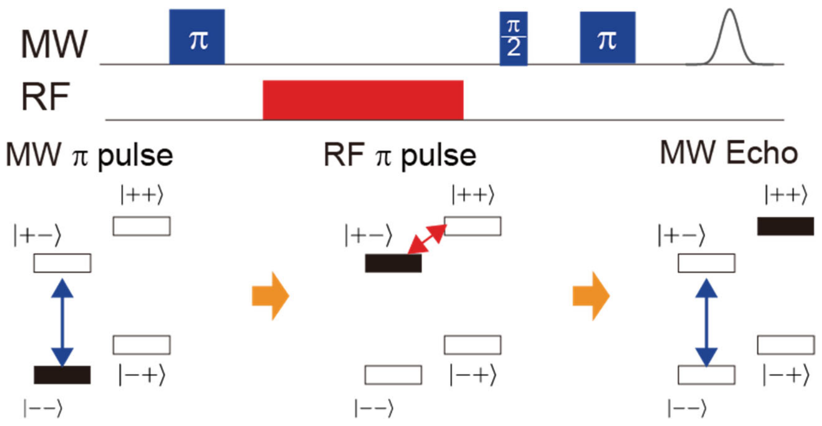

FIG. S3. ENDOR pulse sequence (Top) and polarisation transfers with the above pulse sequence in the quantum system of an electron spin (S = 1/2) and nuclear spin (I = 1/2) (Bottom).

Reference

S1 M. W. Doherty, N. B. Manson, P. Delaney, F. Jelezko, J. Wrachtrup, & L. C. L. Hollenberg, The nitrogen-vacancy colour centre in diamond. *Phys. Rep.* **528**, 1 (2013).

S2 J. E. Mack, A Table of Nuclear Moments, January 1950. *Rev. Mod. Phys.* **22**, 64 (1950).

S3 S. Felton, A. M. Edmonds, M. E. Newton, P. M. Martineau, D. Fisher, D. J. Twitchen, & J. M. Baker, Hyperfine interaction in the ground state of the negatively charged nitrogen vacancy center in diamond. *Phys. Rev. B* **79**, 075203 (2009).

S4 M. Müller, *Introduction to Confocal Fluorescence Microscopy, Second Edition* (SPIE - The International Society for Optical Engineering, Washington, 2006).

S5 A. Schweiger & G. Jeschke, *Principles of pulse electron paramagnetic resonance* (Oxford University Press, New York, 2001).

S6 F. Hoehne, L. Dreher, M. Suckert, D. P. Franke, M. Stutzmann, & M. S. Brandt, Time constants of spin-dependent recombination processes. *Phys. Rev. B* **88**, 155301 (2013).

S7 H. Malissa, M. Kavand, D. P. Waters, K. J. van Schooten, P. L. Burn, Z. V. Vardeny, B. Saam, J. M. Lupton, & C. Boehme, Room-temperature coupling between electrical current and nuclear spins in OLEDs. *Science* **345**, 1487 (2014).
